# Antiurolithic activity of *Origanum vulgare *is mediated through multiple pathways

**DOI:** 10.1186/1472-6882-11-96

**Published:** 2011-10-17

**Authors:** Aslam Khan, Samra Bashir, Saeed R Khan, Anwar H Gilani

**Affiliations:** 1 Department of Pharmacology, Faculty of Pharmacy, University of Karachi, Karachi, Pakistan; 2 Natural Product Research Division, Department of Biological and Biomedical Sciences, Aga Khan University Medical College, Karachi-74800, Pakistan; 3 Centre for the Study of Lithiasis, Department of Pathology, College of Medicine, University of Florida, USA; 4 College of Pharmacy, King Saud University, Riyadh, Saudi Arabia

## Abstract

**Background:**

*Origanum vulgare *Linn has traditionally been used in the treatment of urolithiasis. Therefore, we investigated the crude extract of *Origanum vulgare *for possible antiurolithic effect, to rationalize its medicinal use.

**Methods:**

The crude aqueous-methanolic extract of *Origanum vulgare *(Ov.Cr) was studied using the *in vitro *and *in vivo *methods. In the *in vitro *experiments, supersaturated solution of calcium and oxalate, kidney epithelial cell lines (MDCK) and urinary bladder of rabbits were used, whereas, in the *in vivo *studies, rat model of urolithiasis was used for the study of preventive and curative effect.

**Results:**

In the *in vitro *experiments, Ov.Cr exhibited a concentration-dependent (0.25-4 mg/ml) inhibitory effect on the slope of nucleation and aggregation and also decreased the number of calcium oxalate monohydrate crystals (COM) produced in calcium oxalate metastable solutions. It also showed concentration-dependent antioxidant effect against DPPH free radical and lipid peroxidation induced in rat kidney tissue homogenate. Ov.Cr reduced the cell toxicity using MTT assay and LDH release in renal epithelial cells (MDCK) exposed to oxalate (0.5 mM) and COM (66 μg/cm^2^) crystals. Ov.Cr relaxed high K^+ ^(80 mM) induced contraction in rabbit urinary bladder strips, and shifted the calcium concentration-response curves (CRCs) towards right with suppression of the maximum response similar to that of verapamil, a standard calcium channel blocker. In male Wistar rats receiving lithogenic treatment comprising of 0.75% ethylene glycol in drinking water given for 3 weeks along with ammonium chloride (NH_4_Cl) for the first 5 days, Ov.Cr treatment (10-30 mg/kg) prevented as well as reversed toxic changes including loss of body weight, polyurea, crystalluria, oxaluria, raised serum urea and creatinine levels and crystal deposition in kidneys compared to their respective controls.

**Conclusion:**

These data indicating the antiurolithic activity in Ov.Cr, possibly mediated through inhibition of CaOx crystallization, antioxidant, renal epithelial cell protective and antispasmodic activities, rationalizes its medicinal use in urolithiasis.

## Background

Urolithiasis, the formation of urinary stones, is one of the oldest known diseases. Archaeological findings give profound evidence that humans have suffered from kidney and bladder stones for centuries, even examinations of Egyptian mummies have revealed kidney and bladder stones [1].

It is the third most common problem of the urinary tract with an estimated lifetime risk of 2-5% in Asia, 8-15% in Europe and America and around 20% in the Middle East. It is associated with high rate of recurrence, which is around 10-23% per year, 50% in 5-10 years and 75% in 20 years. Once afflicted, the subsequent relapse rate is increased and the recurrence interval is shortened [2-5]. Moreover, its annual incidences are increasing and also the age of onset is decreasing, perhaps due to change in life styles, diet and climate [6].

Improvement in therapy of kidney stones with modern techniques like extracorporeal shock wave lithotripsy (ESWL), ureteroscopy (URS), and percutaneous nephrolithotomy (PNL), has revolutionized the urological practice but the recurrence in kidney stone is not altered. ESWL is less effective in calcium oxalate monohydrate (COM) and cystine stones than calcium oxalate dihydrate (COD) and uric acid stones [7]. In addition to the high cast and the issues of recurrence with these measures there are multiple side effects, which include renal damage, ESWL induced hypertension, renal impairment, sever haematuria, steinstrasse (multiple small stone blocking ureter), pancreatitis, infection and persistent residual fragments as potential nidus for new stone formation. While these complications can lead to large perfusion of the collecting system, extravasations of irrigating fluid, urosepsis, ureteral injury and delayed bleeding may also occur [8,9]. Pharmacological agents include a limited choice like citrate and thiazide diuretics, which have limited efficacy in addition to their less tolerability [10,11]. On the other hand, there is growing interest of public in herbal medicine, particularly in the treatment of urolithiasis partly because of limited choice in the pharmacotherapy. Moreover, herbal remedies are known to contain multiple constituents, acting through multiple pathways needed in urolithiasis, for example, antispasmodic, diuretic, pain relieving [12,13]. In this study, we evaluated *Origanum vulgare *L for its antiurolithic activity, in an attempt to rationalize its folkloric use in urolithiasis and to see if it exhibits calcium oxalate crystallization inhibitory, antioxidant, renal cell protective, antispasmodic and diuretic activities, which are likely to contribute in its antiurolithic effect. *Origanum vulgare *Linn (family, Lamiaceae) is distributed throughout Asia, Europe and North America [14] and is commonly known as Wild Marjoram and Winter Sweet and locally in Pakistan as Mirzanjosh, Sathra [15,16]. It is widely used in the traditional medicine as lithotriptic, diuretic and antispasmodic along with other medicinal uses, such as stimulant, expectorant, antibacterial, anticancer, anti-inflammatory, antioxidant and laxative [15-17].

## Methods

### Chemicals and reagents

All chemical used were of analytical grade. List and sources of chemical used in the study are provided in the additional file 1.

### Animals

Experiments were performed in compliance with the rulings of the Institute of Laboratory Animal Resources, Commission on Life Sciences, National Research Council [18] and approved by the Ethical Committee for Research on Animals (ECRA) of the Aga Khan University, Karachi, Pakistan.

Wistar rats (180-220 g) of either sex used for this study were sourced locally and housed at the animal house of the Aga Khan University, kept in plastic cages (47 × 34 × 18 cm3) with saw dust (renewed after every 48 hrs), under a controlled temperature of 23-25°C and 12 hrs light-dark cycle. Animals had access to food and water ad-libitum throughout the study. However, food was withdrawn 24 hrs before and during 6 hrs of diuretic study, and while collecting 24 hrs urine samples.

### Plant material and extraction

The plant of *Origanum vulgare *was collected from the Northern Areas of Pakistan, identified by taxonomist Prof. Jhandar shah, Vice chancellor Shaheed Benazir Bhutto University, Khyber Pakhtunkhwa, Pakistan and voucher specimen (OV-PL-02-08-72) was submitted to the herbarium of the Department of Biological and Biomedical Sciences, the Aga Khan University, Karachi. The aerial part of the plant material was cleaned of adulterants and kept soaked for three days in the aqueous-methanol (30:70) with occasional shaking, at room temperature. The filtration was carried out using a muslin cloth and then through Whatman qualitative grade 1 filter paper. This procedure was repeated twice and then all the filtrates obtained were combined and concentrated on a rotary evaporator (RE-111, Buchi, Flawil, Switzerland) accompanied with B-700 recirculation chiller and a water bath model 461 at 40°C to a thick pasty mass called as crude extract (Ov.Cr), yielding approximately 12% [19,20].

### Preliminary phytochemical analysis

The crude extract of *Origanum vulgare*, was screen for the presence of different phytochemical groups such as alkaloids, saponins, coumarins, sterols, terpenes, tannins and flavonoids by using methods followed previous studies [21,22].

### *In vitro *experiments

#### *In vitro *crystallization studies

##### Kinetic study

The effect of the test material on kinetics of calcium oxalate (CaOx) crystallization was determined by the time course measurement of turbidity changes due to the crystal nucleation and aggregation after mixing metastable solutions of calcium (Ca^++^) and oxalate (Ox). Stock solutions of CaCl_2 _(8.5 mM) and Na_2_C_2_O_4 _(1.5 mM), containing 200 mM NaCl and 10 mM sodium acetate were adjusted to pH 5.7 [23]. An aggregometer (Chrono-Log Corporation, USA) devised for platelet aggregation studies based on the measurement of optical density at 620 nm was used to investigate the event of CaOx crystallization [24]. The slopes of nucleation (S_N_) and aggregation phases (S_A_) were calculated using linear regression analysis. Using the slopes, the percentage inhibition was calculated as [(1-Sm/Sc) × 100], where Sm is slope in the presence of modifier; K.Cit or Ov.Cr, and Sc is slope of the control experiment.

##### Incubation study

To determine the effect of incubation on CaOx crystal formation, stock solutions of CaCl_2 _and Na_2_C_2_O_4 _similar to those in the kinetic study were used. CaCl_2 _solutions, containing different concentrations of the Ov.Cr or potassium citrate, were aliquoted (0.5 ml) to the flat-bottomed tubes in a 24 well plate (Iwaki Microplate with lid, Scitech Div., Asahi Techno Glass, Japan). To each of these tubes sodium oxalate (Na_2_C_2_O_4_) solution (0.5 ml) was added [25]. The plates were then incubated in a shaking water bath at the 90 oscillations/min at a temperature of 37°C for 45 minutes. Abundance and the morphology of the crystals in each tube were then observed under inverted microscope (Nikon Corporation, Tokyo, Japan).

### Determination of antioxidant activity

Antioxidant potential of the test materials was estimated *in vitro *by free radical scavenging and lipid peroxidation inhibitory activities.

#### Free radical scavenging activity

For free radical scavenging activity, a 0.1 mM solution of 2,2-Diphenyl-1-picrylhydrazyl (DPPH) radical in methanol was prepared and 1 ml of this solution was added to 3 ml of the test material at different concentrations prepared in methanol [26]. Solutions were incubated for 30 min at room temperature and then absorbance was measured at 517 nm. Decreasing of the DPPH solution absorbance indicates an increase of the DPPH radical-scavenging activity.

#### Lipid peroxidation inhibitory activity

To assess lipid peroxidation inhibitory activity, the kidneys isolated from Wistar rat were homogenized with electric homogenizer (Zero-Max^®^), in ice cold PBS (50 mmol/l, pH 7.4) as described earlier [27].

### Cell lines (MDCK) Experiment

MDCK cells were maintained as sub-confluent monolayers at 37°C in 5% CO_2_. The culture were grown in 75 cm^2 ^Falcon tissue culture flasks in a 1:1 DMEM nutrient mixture and F-12 medium (DMEM/F-12) containing 10% FBS, 2% streptomycin/penicillin, pH 7.4. The medium was changed every 3 to 4 days.

#### XTT assay for cell viability

Media was aliquoted to designated wells, containing confluent MDCK cells, of a 96 well plate. The XTT Cell Viability Assay Kit was used to determine the cell viability. Cells were incubated with and without (Control) plant extracts for 24 hrs. Then 50 μL of the working activation solution, prepared by mixing of one bottle of XTT solution and one of the activation reagent PMS supplied with kit, was added to all the samples and control, and incubated for 2-4 hrs. Optical density absorbency was read at 450-490 nm on a Bio-Rad 3550 microplate reader (Bio-Rad, Hercules, CA).

#### Lactate Dehydrogenase (LDH) Release

Media was aliquated to designated wells of a 96 well plate. The CytoTox 96 Non-Radioactive Cytotoxicity assay kit was used to determine LDH percent release. Substrate (supplied with kit) was added to all samples, positive control (MDCK cells lysed with lysis solution-supplied with kit), and blanks (acclimazation media). The plate was incubated at room temperature for 30 minutes in the dark. Stop solution (supplied with kit) was added to all samples, positive control, and blanks. Optical density absorbency was read at 490 nm on a Bio-Rad 3550 microplate reader (Bio-Rad, Hercules, CA).

### Antispasmodic activity

Antispasmodic activity of the plant extract was evaluated against carbachol (CCh) and high K^+ ^(80 mM)-induced contractions in strips of rabbit urinary bladder [28]. The whole urinary bladder of rabbit was dissected out, and divided into 3-4 vertical strips. Each preparation was mounted in a 10-20 ml tissue bath containing Kreb's-Henseleit solution, maintained at 37°C and continuously aerated with carbogen. A tension of 1 g was applied to each tissue throughout the experiment. Isometric responses were recorded on a Grass Model Polygraph and/or Power lab 4/24 data acquisition system attached to computer running Chart 5.3 software (AD Instruments, Sydney, Australia). The tissues were allowed to equilibrate for a period of about 1 hr before the addition of any drug, during which the tissue was washed with fresh bathing fluid at an interval of every 15-20 minutes.

Following the equilibrium, preparations were stabilized, by repeatedly treating with 1 μM CCh, until constant responses were recorded. Then the spasmolytic activity of the plant extract was determined by adding the plant extracts on CCh or high K^+^-induced sustained contraction in rabbit bladder, in a cumulative fashion to obtain concentration-dependent inhibitory response [29]. IC_50 _values (concentration causing 50% inhibition) were calculated from these curves and calculated as a measure of spasmolytic potency of the relaxant drugs.

#### Calcium channel blocking activity

The concentration-response curves (CRCs) of Ca^++ ^were constructed in the absence and presence of increasing concentrations of test drug to confirm the Ca^++ ^antagonist action of the test substance. The tissue was allowed to stabilize in normal Kreb's-Henseleit solution, which was then replaced with Ca^++^-free Kreb's-Henseleit solution containing EGTA (0.1 mM) for 30 min in order to remove Ca^++ ^from the tissues. This solution was further replaced with K^+^-rich and Ca^++^-free Kreb's-Henseleit solution. Following an incubation period of 30 min, CRCs of Ca^++ ^were constructed in the absence and the presence of different concentrations of the test materials [30]. Isometric responses were recorded on a Grass Model 7 Polygraph and/or Power lab 4/24 data acquisition system attached to computer running Chart 5.3 software (AD Instruments, Sydney, Australia).

### *In vivo *experiments

#### Determination of diuretic activity

The diuretic activity of the test material was studied on Wistar rats of either sex (180-220 g) as described previously [31]. Animals were divided with matched body weight and sex into groups of 6 animals each. Normal and positive control groups were given by gavages saline (20 ml/kg) and standard diuretic drug: hydrochlorothiazide (HCT), 10 mg/kg of body weight, respectively. The rest of the groups were given different doses of the test material dissolved in saline. Subsequently, the animals were placed individually in metabolic and diuretic cages. The urine was collected in graduated cylinders for 6 hrs at 2 hrs intervals. Total urine excreted out was collected and the volume was determined.

#### Study on animal model of urolithiasis

Antiurolithic activity of the test material was determined using animal model of CaOx urolithiasis [32,33]. Male Wistar rats (weighing 180-220 g) were divided with matched body weights into groups of 6-8 animals each, which were then randomly selected to receive various treatments for preventive and curative study.

##### Preventive study model

To study the preventive effect of the test material, rats serving as normal control, received intra-peritoneal (i.p.) injections of normal saline (2.5 ml/kg), once in 24 hrs. Whereas, untreated group received i.p. injection of normal saline (2.5 ml/kg) along with renal CaOx crystal deposits inducing (lithogenic) treatment for 21 days. The renal CaOx deposits induction was achieved by giving water containing 0.75% (w/v) ethylene glycol (EG) and 1% (w/v) ammonium chloride (AC) for 5 days, following this the water supply was switched to 0.75% EG alone [32,33]. Treated groups received i.p. injection of the extract dissolved in saline once in 24 hrs and simultaneously received crystal deposits inducing treatment similar to the untreated group. Based on the medicinal use and literature available on *Origanum vulgare*, where doses of 20-60 mg/kg have been used in rats [34,35], we used log doses 10 and 30 mg/kg for its diuretic and antiurolithic effect, optimized in our pilot study. Animal weight and activity were regularly monitored to assess their overall health and those looking lethargic or who have lost excessive weight were excluded from the study. Water intake was determined and 24 hrs urine samples were collected immediately before the onset and at the end of total 21 days of treatment, for which animals were housed individually in metabolic and diuretic cages. The 3 hrs morning urine for crystalluria study was also collected before collecting 24-hrs urine at the end of 21 days of treatments. The number of the crystals/mm^3 ^was counted under light microscope using haemocytometer.

##### Curative study model

In the study for the curative effect, CaOx deposits were induced in the kidneys of rats in both untreated and treated groups with EG (0.75%) and AC (1%) treatment for 21 days, following the plan as given with the study on the preventive effect. Thereafter, lithogenic treatment was withdrawn and treatment with vehicle and the test material was respectively started to the untreated and the treated groups for another period of 14 days [33]. Normal control, received no treatment for the first 21 days study period, but received i.p. injections of normal saline (2.5 ml/kg), during the next 14 days. Animal weights and their activity were regularly monitored, whereas, 24 hrs urine samples were collected immediately before and after the crystals deposits induction (lithogenic) treatment and at the end of total treatment period (35 days). 3-hrs urine sample was collected after both 21 and 35 days.

Following volume and pH determination, 24 hrs urine samples were stored at -20°C until analyzed. Blood was collected through cardiac puncture from animals under ether anaesthesia for serum separation in order to assess serum creatinine and blood urea nitrogen (BUN). Animals were sacrificed and the kidneys were fixed in 10% neutral buffered formalin, processed, embedded in paraffin wax, sectioned at 5 μm and stained with Haematoxylin and Eosin (H & E) and by Pizzolato's method, for calcium oxalate crystals [36], for microscopic examination.

### Calcium oxalate crystal deposition in kidney

Crystal distribution within the kidneys was determined by using the semiquantitaive scoring by the methods used by Vanachayangkul et al., [37]. Briefly the crystal deposits in stained sections with visible in a field of 10× magnification were counted and severity grades were assigned as 0 = < 1 crystals, 1 = ≤ 10, 2 = ≤ 30, 3 = ≤ 50, 4 = ≤ 75 and 5 = > 75 crystals. Most of the crystals were located in the outer modularly and cortical region of the kidney.

### Biochemical analysis of urine and serum

Urine samples for Ox, Ca^++ ^and Mg^++ ^, citrate, and uric acid (UA) and serum samples for creatinine and BUN contents were determined by using commercially available kits (Company names for kits are given in the additional file), while urinary inorganic phosphate and protein were determined by using the molybdenum blue reaction [38] and Lowry's [39] method respectively.

### Data Analysis

The data expressed are mean ± standard error of mean (SEM) and the median effective concentration (EC_50 _value) with 95% confidence intervals (CI). All statistical comparisons between the groups are made by means of t- test (comparison between two groups) or One Way Analysis of Variance (ANOVA) with post hoc Dunnett's test. *p *value less than 0.05 is regarded as significant. CRCs were analyzed by non-linear regression using GraphPad Prism (GraphPad Software, San Diego, CA, USA).

## Results

### Phytochemical Screenings

The phytochemical test showed the presence of saponins, alkaloids, coumarins, sterol, terpenes, flavonoids and tannins in Ov.Cr.

### *In vitro *Experiment

#### Effect on in vitro crystallization

##### Kinetic study

**Figure 1:**
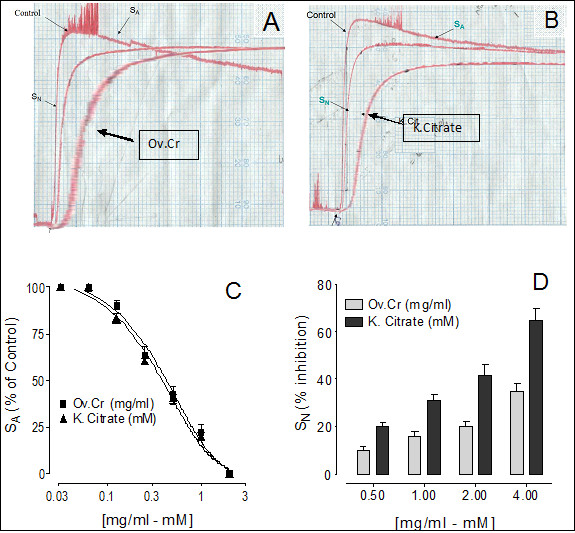
**Calcium Oxalate crystallization study**. Effect of *Origanum vulgare *(Ov.Cr) and Potassium Citrate (k-Cit) on calcium oxalate crystallization. (A) and (B) are the typical tracing of the control and in the presence of Ov.Cr and potassium citrate. Panel (C) is concentration-response curves of Ov.Cr and potassium citrate on S_A _of the turbidity curves, while (D) shows the % inhibition on the S_N_. Symbols shown are mean ± S.E.M. (n = 3). S_N _and S_A _represent slope of nucleation and slop of aggregation respectively.

Effect of Ov.Cr on various phases of CaOx crystallization as determined by time course measurement of turbidity under standard conditions (4.25 mM Ca^++ ^and 0.75 mM Ox) is given in Figure 1, which shows typical tracing (Figure 1A and 1B) of the experiment in the presence of Ov.Cr and potassium citrate. In the control curve, the initial rise in turbidity; the *nucleation phase*, on attaining its maximum after about 150 ± 15 sec, followed by a slow decrease; the *aggregation phase*. Ov.Cr inhibited the S_A _with a median inhibitory concentration of 0.47 mg/ml (0.31 to 0.69; 95% CI), similar to potassium citrate which causes inhibition with IC_50 _value of 0.42 mM (0.31 to 0.58; 95% CI) as shown in Figure 1C. Ov.Cr caused 10 ± 1.73, 16 ± 2.3, 20 ± 1.7 and 35 ± 2.8% inhibition of S_N _at the concentration of 0.5, 1, 2 and 4 mg/ml, while the citrate caused 20 ± 1.7, 31 ± 2.31, 42 ± 4.0 and 65 ± 4.2% inhibition at a concentration of 0.5, 1, 2 and 4 mM, respectively (Figure 1D).

##### Incubation study

**Figure 2:**
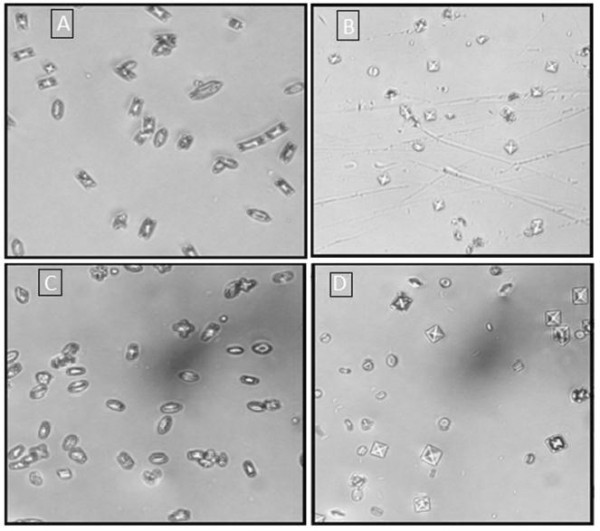
**Calcium Oxalate incubation study**. Representative photographs, under inverted microscope (200x), of CaOx crystals developed in the metastable solutions in the absence (A and C) and in the presence of crude extract of *Origanum vulgare *(Ov.Cr) 2 mg/ml (B) and 2 mM K-Citrate (D).

In the incubation study, mixing the metastable solutions of Ca^++ ^and Ox resulted in the formation of CaOx crystals, predominately of the dumbbell shaped calcium oxalate monohydrate (COM). Ov.Cr caused a concentration-dependent (1-4 mg/ml) decrease in the CaOx crystal formation (Figure 2A &2B) and also decreased the number and size of COM crystals; similarly citrate also decreased the number and size of crystals formed (Figure 2C &2D).

#### Antioxidant activity

##### Free radical scavenging activity

**Figure 3:**
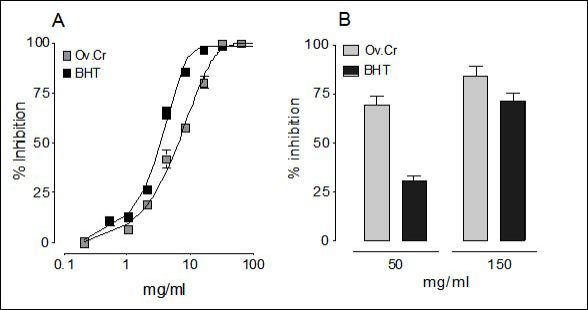
**Antioxidant activity**. Concentration-response curves of the free radical scavenging activity of the butylated hydroxytoluene (BHT) and Ov.Cr, while bar-chart (B) representing lipid peroxidation inhibitory activity of two different concentrations of Ov.Cr and BHT. Inhibition is measure as % of the respective control experiments. The values shown are mean ± SEM (n = 3).

Ov.Cr caused inhibition of DPPH free radical with IC_50 _value of 6.28 μg/ml (5.41 - 7.27; 95% CI), while the control drug butylated hydroxytoluene (BHT) inhibited DPPH with IC_50 _value of 3.41 μg/ml (3.16 - 3.68; 95% CI) as shown in the Figure 3A.

##### Lipid peroxidation inhibitory activity

**Figure 4:**
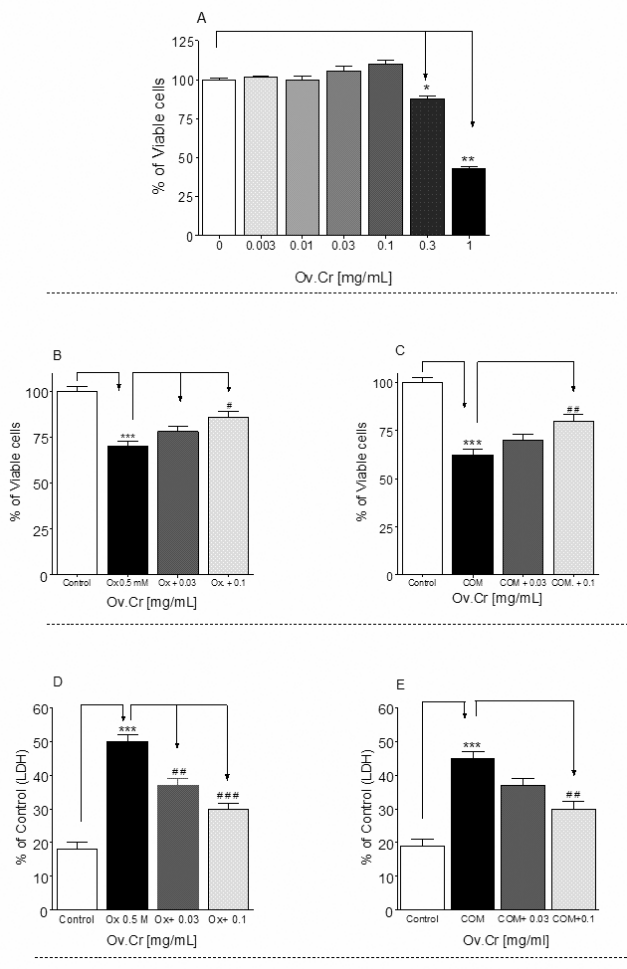
**Effect on MDCK cells**. Effects of various concentrations of Ov.Cr on MDCK cell survival in acclimatization media (A). B and C show the protective effect of Ov.Cr after exposure to 0.5 mM oxalate or 66 mg/cm^2 ^COM respectively. While (D) and (E) shows the percent increase in LDH release against control by MDCK cells exposed Ox. (0.5 mM) and COM (66 μg/cm^2^) for 24 hrs. Data shown are mean ± SEM of two separate experiments with 3 independent replicates. * *p *< 0.05, ** *p *< 0.05 and *** *p *< 0.001

Ov.Cr inhibited the *in vitro *lipid peroxidation induced in rat kidney homogenate by 69.3 ± 4.7% and 84.1 ± 5.0% (Figure 4B), while BHT caused 30.8 ± 2.6 and 71.6 ± 3.8% inhibition of lipid peroxidation at 50 and 150 μg/ml, respectively (Figure 3B).

#### Effect on Kidney Epithelial Cell Lines (MDCK)

##### Effect on cell viability

Ov.Cr had no toxic effect on MDCK cells up to 0.1 mg/ml. However, it significantly (*p *< 0.01) reduced the cell viability at higher concentrations (Figure 4A).

##### Effect Ov.Cr on MDCK cells after exposure to Oxalate and COM crystals

The cell viability was decreased (*p *< 0.001) after exposure to 0.5 mM Ox or 66 μg/cm^2 ^of COM. However, after the co-exposure of Ov.Cr, the cell viability was mildly increased at 0.03 mg/ml while significantly (*p *< 0.01) increased as compared to Ox or COM at a concentration of 0.1 mg/ml (Figure 4B and 4C).

##### Effect of Ov.Cr on cell membrane damage

LDH release was significantly increased (*p *< 0.001) after exposure to 0.5 mM Ox or 66 μg/cm^2 ^COM vs. untreated control. However, co-exposure of Ov.Cr (0.1 and 0.3 mg/ml) significantly decrease the LDH release (Figure 4D and 4E).

#### Effect on Urinary bladder

**Figure 5:**
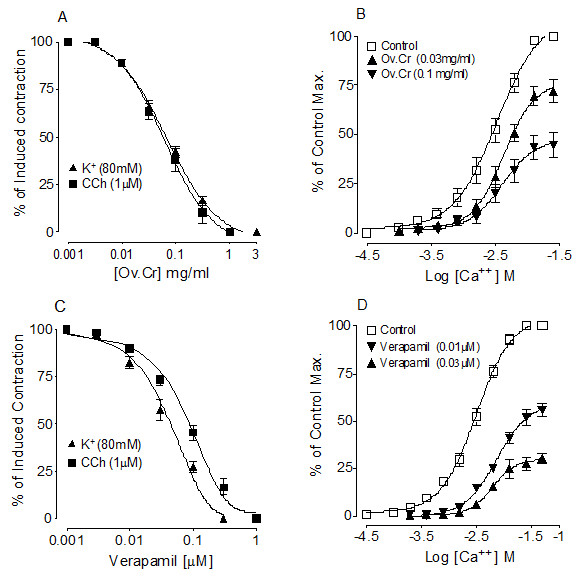
**Antispasmodic effect**. Concentration-response curves of crude extract of Ov.Cr (A) and verapamil (B) on K^+ ^(80 mM) and CCh (1 μM)-induced contractions and the concentration-response curves of Ca^++ ^constructed in the absence and presence of increasing concentrations of Ov.Cr (C) and verapamil (D) in isolated rabbit urinary bladder. The symbols represent mean ± SEM (n = 4-6).

Ov.Cr caused concentration-dependent inhibition of both CCh (1 μM) and high K^+ ^(80 mM)-induced contractions in rabbit urinary bladder preparations (Figure 5A) with an EC_50 _values of 0.061 (0.04-0.08) and 0.068 mg/ml (0.05-0.08) respectively. Similarly, verapamil was found to be more potent against the K^+ ^than the CCh-induced contraction. It relaxed both CCh and K^+^-induced contractions (Figure 5C) with IC_50 _values of 0.08 μM (0.07-0.10) and 0.04 μM (0.03-0.5) respectively. Ov.Cr (0.03-0.1 mg/m1) caused rightward shift of the Ca^++ ^CRCs accompanied by suppression of the maximum contractile effect, similar to that caused by verapamil (0.01-0.03 μM), as shown in Figure 5B and 5D.

### *In vivo *experiments

#### Diuretic effect

**Figure 6:**
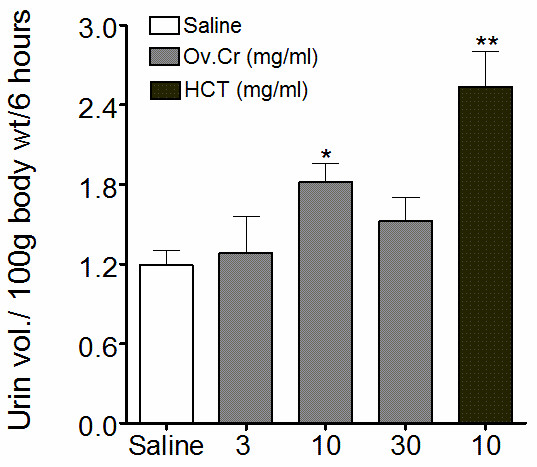
**Diuretic effect**. Effect of the Ov.Cr and hydrochlorothiazide (HCT) on urine volume collected in 6 hrs (values shown are means ± SEM, n = 6-8). * *p *< 0.05, ** *p *< 0.01

Ov.Cr increased significantly (*p *< 0.05) the urine output in Wistar rats at the dose of 10 mg/kg, while there was no affect seen at lower (3 mg/kg) or higher dose (30 mg/kg). HCT (10 mg/kg) was used as reference drug, which significantly (*p *< 0.01) increased the urine output (Figure 6).

#### Effect on animal model of Urolithiasis

##### Preventive effect

In preventive study, all the parameters, like body weights, 24 hrs water intake, urine volume, urinary pH and composition, recorded before the treatment were not significantly different among the groups. The parameters recorded at day 0 and at the end of 3 weeks of treatment period are listed in the additional file 2.

**Figure 7:**
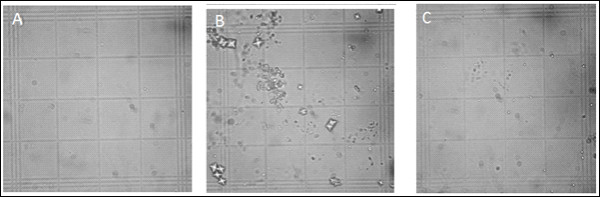
**Images of Crystalluria**. Images of calcium oxalate crystals in 3 hrs morning urine collected from Normal control (A), Lithogenic Control (B) and treated with crude extract of *Origanum vulgare*(Ov.Cr) (C), under light microscope at 400× magnification.

In the 3-hrs morning urine sample of rats, significantly more and bigger CaOx crystals mostly of COD were observed in lithogenic group as compared to the saline group. Whereas, Ov.Cr significantly reduced urinary crystal count as well as decreased crystal size (Figure 7). At the end of the treatment a significant (*p *< 0.01 vs. Normal) loss in body weights was caused by the EG and AC consumption in the lithogenic group as compared to the normal saline group. The co-administration of Ov.Cr (10-30 mg/ml) prevented the loss in body weights of rats (*p *< 0.01 vs. lithogenic group). 24 hrs urine volume and water intake were higher (*p *< 0.01) in the lithogenic group compared to that of normal saline animals. Urine pH was also reduced, though not to a significant extent. A co-treatment with Ov.Cr significantly reduced (*p *< 0.05) polyurea and water intake compared to lithogenic group. Similarly, oxalate excretion was significantly increased (*p *< 0.01) in lithogenic animals, whereas Ca^++ ^excretion was decreased (*p *< 0.05). Urine contents of citrate, phosphate, UA, Mg ^2+^, Na^+ ^and K^+ ^did not alter to a significant level. Co-administration of Ov.Cr (10-30 mg/kg) to lithogenic group significantly (*p *< 0.05) decreased oxalate excretion, whereas urinary excretion of citrate and Ca^2+ ^was significantly increased (*p *< 0.05). EG treatment caused impairment of renal functions of the untreated rats (lithogenic group) as evident from total protein loss and raised BUN and serum creatinine (*p *< 0.05), which were prevented in the animals treated with Ov.Cr. (please see additional file 2).

**Figure 8:**
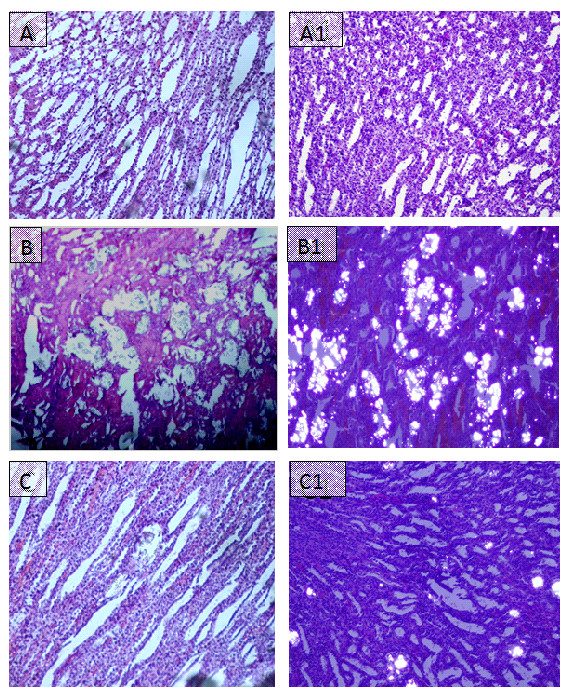
**Microscopic images of Kidney sections in preventive study**. Representative microscopic images of the H and E stain of the kidney sections from normal (A), Lithogenic group (B) and Treated (C) with Ov.Cr. A1, B1 and C1 show the polarized images of the sections.

**Figure 9:**
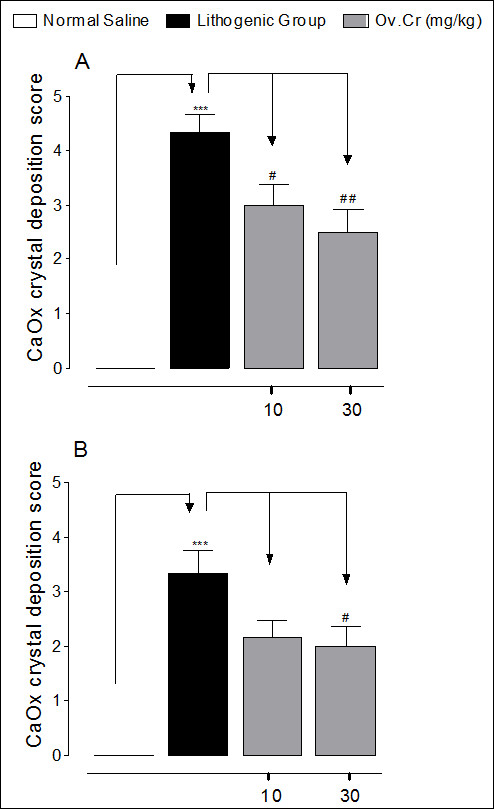
**Crystals deposits score in Kidney sections of preventive and curative study**. Calcium oxalate crystal deposition score after treatment with 0.75% EG, 1% NH_4_Cl (lithogenic group), Ov.Cr 10 and 30 mg/kg in preventive (A) and curative (B) study model. Severity grade were assigned as 0 = < 1 crystals, 1 = ≤ 10, 2 = ≤ 30, 3 = ≤ 50, 4 = ≤ 75 and 5 = > 75 crystals; data are expressed as mean ± SEM. * *p *< 0.05, ** *p *< 0.05 and *** *p *< 0.001 vs. Normal, ^# ^*p *< 0.05, ^## ^*p *< 0.01 and ^### ^*p *< 0.001 vs. Lithogenic group.

Kidneys excised from lithogenic group were enlarged. Histological preparations of kidneys of normal saline group did not show any crystalline deposits, whereas, a high score of crystalline deposits was observed in all regions of kidneys in the lithogenic group. However, Ov.Cr treatment significantly lowered the CaOx crystal deposits (*p *< 0.5) as compared to untreated (lithopgenic) group (Figure 8 and 9A).

##### Curative effect

In the curative study, all the parameters were recorded before start of the treatment (day 0), after 3 weeks of the crystals deposits induction, and then two weeks after the withdrawal of the crystals deposition treatment (0.75% EG + 1% AC in drinking water). The parameters recorded for the curative study are given in the Additional file 3.

The groups, which consumed the lithogenic treatment for the first 21 days, developed the lithogenic parameters as compared to the normal saline group like in the preventive study. This was suggested by a net loss in the body weights, a significant increase in 24 hrs water intake, urine volume, oxalate, uric acid, total protein, and decreased urinary pH and Ca^++ ^contents, impaired renal function suggested by increased serum creatinine, BUN and total urinary protein loss (Additional file 3). Post-induction treatment with Ov.Cr (10 and 30 mg/kg) reversed the loss in body weights, impaired urinary and serum functions, crystalluria and deposition of crystals in the kidney more quickly than the control group (Additional file 3).

**Figure 10:**
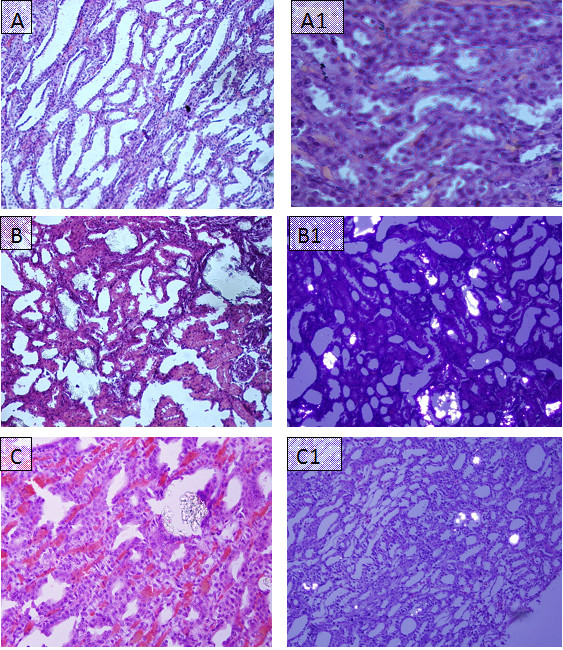
**Microscopic images of Kidney sections in curative study**. Representative microscopic images of the H and E of the kidney sections from normal (A), Lithogenic group (B) and Treated (C) with Ov.Cr. A1, B1 and C1 show the polarized images of the sections.

After two weeks of Ov.Cr treatment in the curative study, renal CaOx crystal deposits were found in 4 out of 6 rats. However, they were significantly less (*p *< 0.5) than the untreated group (Figure 9B and Figure 10).

## Discussion

In view of the medicinal uses of *Origanum vulgare *in urolithiasis, we evaluated its crude extract for the possible antiurolithic effect along with antioxidant, antispasmodic and diuretic activities using the *in vitro *assays and *in vivo *rat models.

In this study, the plant extract inhibited the CaOx crystal nucleation and aggregation in a concentration-dependent manner, similar to citrate, a well-known inhibitor of CaOx crystallization and clinically used for the management of urolithiasis [40]. Similarly, in the incubation study, Ov.Cr caused a decrease in crystal count and transformed COM to COD crystal like that of citrate and Mg^2+ ^[41]. COM crystals are considered to be more harmful than COD because of their tendency to attach with the membrane to form aggregates [42] and are more likely to attach with the kidney epithelial cells than CaOx dehydrate, resulting in the formation of kidney stones [42,43]. Calcification is a multifactorial phenomenon [44], arising as a result of a cascade of events initiated by supersaturation, including crystal nucleation, growth, aggregation and retention [45]. Although supersaturation is not the only critical step involved in the formation of kidney stones as several studies have identified many inhibitors of calcium oxalate and calcium phosphate crystallization including ionic or macromolecules [46], yet it is the prerequisite for the crystals formation in the urinary tract [47]. Various crystal inhibitors like potassium-sodium citrate and magnesium oxide have been shown to decrease the saturation of CaOx and inhibit crystal nucleation, growth and aggregation and reduced crystallization in urine of stone forming patients [48]. Interference with crystal growth and aggregation therefore seems a possible therapeutic strategy for the prevention of recurrent stone disease. Ov.Cr inhibits CaOx crystal nucleation and aggregation along with decrease in count and morphological change, from COM to COD, in crystals. These results indicate the presence of CaOx crystal aggregation inhibitory constituent(s) in the plant.

Animal and cellular studies have revealed that oxalate, calcium oxalate and hydroxy apatite crystals cause injury to kidney cells [49,50], caused by the production of reactive species (ROS) which is considered to be the risk factor for the crystallization and crystals deposition in the kidney by promoting crystal nucleation, aggregation, retention and stone development [49,51]. Antioxidants such as vitamin E, catechin and selenium have been shown to protect against oxidative injury by oxalate and crystal deposition [52,53]. When studied for its antioxidant activity, Ov.Cr caused scavenging of DPPH free radical and inhibited ferrous-ascorbate-induced lipid peroxidation of rat kidney homogenate similar to BHT, a standard antioxidant [54], confirming its antioxidant activity [35,55,56].

Cytoprotective effect of the plant was confirmed when pre-treatment of the normal kidney epithelial (MDCK) cells with Ov.Cr significantly increased the survival rate and reduced the LDH, a marker of cell membrane damage [57], release of MDCK cells exposed to Ox and COM crystals. This protective effect could be the result of its antioxidant activity.

Antispasmodics are more commonly used in the medical expulsive therapy (MET) for the kidney stones [58]. Administration of alpha-adrenoreceptor antagonists or Ca^++ ^channel blockers enhances the expulsion rate of crystals and reduces colic event [59]. Therefore, we evaluated the antispasmodic effect of Ov.Cr, and studied its relaxant effect against high K^+ ^(80 mM) and CCh (1 μM) induced contraction, using rabbit urinary bladder strips. High K^+ ^(> 30 mM) is known to cause smooth muscle contraction though opening of voltage depended L-type calcium channels, thus allowing influx of extracellular Ca^++ ^resulting in the contraction of the smooth muscles [20,60] and a substance that relax the high K^+ ^induced contraction is considered to be CCB [61]. Whereas, CCh is a cholinergic drug, which can induce contraction in urinary bladder through activation of muscarinic receptors, predominately M_3 _subtype [62]. Ov.Cr relaxed the high K^+ ^and CCh induced contraction like that of verapamil, a standard CCB [63], indicating CCB activity, which was confirmed when pre-treatment of the tissue with the plant material shifted the Ca^++ ^CRC to the right with suppression of the maximum response, like that of verapamil.

In the *in vivo *experiment, Ov.Cr caused significant increase in urine output at the dose of 10 mg/kg similar to that caused by HCT, a standard diuretic, while, the administration of next higher dose (30 mg/kg) did not caused any significant change in the diuretic effect, compared to the saline control, which could be due to the co-existence of anti-diuretic component(s) in Ov.Cr, as plant extract may exhibit multiple therapeutic activities probably on account of having a mixture of phytochemicals. The presence of synergistic and/or side-effect neutralizing effect in plants is known to exist [12], which is probably meant by nature not to allow the pharmacological effect go beyond a certain limit, beyond which it could have been harmful. Diuretics increase urine volume, which results in the reduction of supersaturation of crystals forming salts and also help in the expulsion of already formed crystals [64,65].

The antiurolithic effect of Ov.Cr was evaluated on the most commonly applied EG-induced model for urolithiasis [66-68] and male Wistar rats, which develop changes in urine electrolytes and CaOx super-saturation to a greater extent due to their greater sensitivity to EG toxicity [69], were used.

In this study, hyperoxaluria was induced by administration of EG (0.75% in drinking water) for 21 days and AC (1%) was given only for the first 5 days, as administration of AC for more than 5 days led to extreme weight loss and ultimately death of the rats [37].

In the preventive study, administration of EG and AC resulted in the increase in crystalluria with larger crystals due to hyperoxaluria, increase in water intake and urine output, which might be due to the renal impairment [68], as there was significant increase in serum creatinine, blood urea nitrogen and total protein loss in lithogenic group as compared to normal, which has been restored by the Ov.Cr treatment. Consistent with some previous reports, crystals deposition by hyperoxaluria caused an increase in oxalate and decrease in Ca^2+ ^excretion in the lithogenic group [70,71], which was prevented by Ov.Cr. There was hypertrophy and extensive calcium oxalate crystal deposition in kidneys of lithogenic group. The renal tubules were markedly dilated, which might be due to the obstruction in distal renal tubular flow by large crystals [68]. Several *in vivo *and *in vitro *studies have demonstrated that hyperoxaluria, a major risk factor for calcium oxalate nephrolithiasis, results in production of superoxide and hydroxyl free radicals causing oxidative stress, cell membrane rupture and cell death [53,72], and leads to CaOx crystal adherence and retention in renal tubules [53,73]. This can be speculated that the inhibitory effect of Ov.Cr on calcium oxalate crystal deposition in renal tubules could have also been caused by its antioxidant activity.

In curative study, withdrawal of lithogenic treatment after 21 days evoked a spontaneous recovery of nephrolithic animals in the untreated groups. However, Ov.Cr enhanced the spontaneous recovery in the treated group than the untreated group, which was clearly shown by the gain in body weight, significant decrease in urinary oxalate, renal crystal deposition and improvement in renal functions compared to the lithogenic group.

Phytochemical screening revealed the presence of saponins, alkaloids, coumarins, sterol, terpenes, flavonoids and tannins. Different activities observed in the crude extract might be due to the presence of these phytochemicals. For example, flavonoids are known to possess antispasmodic and Ca^++ ^channel blocking [74,75], antioxidant [76] and diuretic [77] activities. Saponins are known to possess anti-crystallization property by disaggregating the suspension of mucoproteins, promoters of crystallization [9]. However, the contribution of other phytochemicals accounting for the reported activities cannot be rule out.

The plant showed antiurolithic activity both in the *in vitro *and *in vivo *models in addition to its antioxidant, renal epithelial cell protective, antispasmodic and diuretic activities reported in this study, all of which could be beneficial in urolithiasis. The plant is also reported to possess anti-inflammatory [78] and antimicrobial [79] activities, which could also be supplementing its beneficial effect, as the infection and inflammation are likely to be associated with urolithiasis process. Thus, the herbal remedies known to contain multiple activities offer therapeutic potential particularly in the urolithiasis, where multiple targets are needed. A few studies on the effectiveness of herbal remedies in urolithiasis exists, such as *Hernaira hirsute, Phylanthus niruri *and *Hibiscus sabdariffa*, which showed promising results in the management of urolithiasis [8]. However, most of these studies were using the *in vitro *and/or *in vivo *studies with limited advancement in the possible mechanisms of their effectiveness. In this study, we used both the *in vivo *and *in vitro *models along with multiple activities, as mentioned above, giving comprehensive information on the pharmacological basis for its effectiveness in urolithiasis. The plant being of edible nature with a long history of medicinal use is considered to be relatively safe, however, detailed studies on its safety profile is needed before recommending for clinical use.

## Conclusion

Together these data suggest that the presence of antiurolithic effect in *Origanum vulgare *against renal calcium oxalate crystal deposits is mediated possibly through a combination of CaOx crystal inhibitory, diuretic, antioxidant, antispasmodic, epithelial cell protective, hypocalciuric and hypercitrauric effects thus acting on multiple sites. This study rationalizes its medicinal use in the treatment of urolithiasis.

## Competing interests

The authors declare that they have no competing interests.

## Authors' contributions

AK carried out the draft, experimental work, data collection and evaluation, literature search and manuscript preparation. SB helped in the experimental study designing and corrected the manuscript for publication. SRK helped in the cell culture experiments and refined the manuscript for publication. AHG supervised the work and refined the manuscript for publication. All authors read and approved the final manuscript.

## Pre-publication history

The pre-publication history for this paper can be accessed here:

http://www.biomedcentral.com/1472-6882/11/96/prepub

## Supplementary Material

Additional file 1 **Chemicals and reagents**. List of names and sources of the chemicals and reagents used in the study. Click here for file

Additional file 2 **Table S1**. Various parameters from different groups of rats recorded after 21 days, to study the preventive effect in animal model of urolithiasis Click here for file

Additional file 3 **Table S2**. Various parameters from different groups of rats, recorded after 21 and 35 days, to study the curative effect in animal model of urolithiasis Click here for file
