# Supplementary material for: Antiurolithic activity of Origanum vulgare is mediated through multiple pathways
Source: BMC Complement Altern Med. 2011 Oct 17;11:96. doi: 10.1186/1472-6882-11-96 (PMC3222619; doi:10.1186/1472-6882-11-96)
Supplement: Additional file 1 — Chemicals and reagents. List of names and sources of the chemicals and reagents used in the study. [file 1472-6882-11-96-S1.DOC]

## Chemicals and reagents

The following chemicals were obtained from the sources specified: ammonium chloride, 1,1-diphenyl-2-picrylhydrazyl (DPPH), bovine serum albumin, butylated hydroxyl toluene (BHT), calcium oxalate monohydrate crystals (COM), carbachol (carbamylcholine chloride, CCh), disodium oxalate (Na_2_C_2_O_4_), ethylene glycol (EG), ferrous sulfate heptahydrate (FeSO_4_.7H_2_O), Hydrogen peroxide (H_2_O_2_), n-butanol and petroleum spirit (Merck, Darmstadt, Germany). Potassium citrate tribasic hydrate, thiobarbituric acid, potassium chloride, verapamil hydrochloride, thymol, trichloroacetic acid, streptomycin/penicillin (Sigma Chemical Company, St. Louis, MO, USA). Madin Darby Canine Kidney (MDCK) cells (Cat.# CRL-34; Manassa, VA, U.S.A), mixture of modified Eagle’s medium (DMEM), F-12 medium (DMEM/F-12) and fetal bovine serum (FBS) (Gibco BRL, Grand Island, NY, USA), 96 well plate (Fisher Scientific, Norcross, GA Cat # 21-377-205) Kits used in this study for XTT was from Biotium, Inc., Haward, Ca, USA Cat # 30007 and for LDH release was from Fisher Scientific, Norcross, GA, Promega Cat # PR-G1780. Other kits for the determination of calcium, magnesium, and blood urea nitrogen were supplied by Randox Laboratories Ltd. Ardmore, Diamond Road, Crumlin, Co. Antrim, UK. Oxalate estimation was done by the kit from Trinity Biotech Plc, IDA business park, Bray, Co. Wicklow, Ireland and citrate estimation by the kit purchased from R-Biopharm AG, D-64293 Darmstadt. Reagents of histopathology: Eosin, Hematoxylin, xylene, paraffin wax, Chemicals used for making physiological salt solutions were: calcium chloride (CaCl_2_), ethylenediamine tetraacetic acid (EDTA), glucose, magnesium chloride (MgCl_2_), magnesium sulfate (MgSO_4_), potassium chloride (KCl), potassium dihydrogen phosphate (KH_2_PO_4_), sodium bicarbonate (NaHCO_3_), sodium dihydrogen phosphate (NaH_2_PO_4_) from Merck, Darmstadt, Germany, while from Sigma Chemical Co, St Louis, MO, USA and sodium chloride (NaCl) from BDH Laboratory supplies, Poole, England. The chemicals used in phytochemical analysis include: acetic anhydride, aluminum chloride, ammonium hydroxide, benzene, chloroform, Dragendorff's reagent, ferric chloride (Sigma Chemical Co, St Louis, MO, USA), hydrochloric acid and petroleum ether (BDH Laboratory supplies, Poole, England). All the chemicals used were of analytical grade available.
